# Electrochemically Deposited MoS_2_ and MnS Multilayers on Nickel Substrates in Inverse Opal Structure as Supercapacitor Microelectrodes

**DOI:** 10.3390/mi14020361

**Published:** 2023-01-31

**Authors:** Sheng-Kuei Chiu, Po-Yan Chen, Rong-Fuh Louh

**Affiliations:** Department of Materials Science and Engineering, Feng Chia University, Taichung 407102, Taiwan

**Keywords:** supercapacitor microelectrodes materials, molybdenum disulfide (MoS_2_), manganese sulfide (MnS), electrophoretic self-assembly (EPSA), electrochemical deposition (ECD), inverse opal structure (IOS), photonic crystals (PhCs), PS microspheres

## Abstract

High-dispersion polystyrene (PS) microspheres with monodispersity were successfully synthesized by the non-emulsification polymerization method, and three-dimensional (3D) photonic crystals of PS microspheres were fabricated by electrophoretic self-assembly (EPSA). The metal nickel inverse opal structure (IOS) photonic crystal, of which the structural thickness can be freely adjusted via electrochemical deposition (ECD), and subsequently, MnS/MoS_2_/Ni-IOS specimens were also prepared by ECD. Excellent specific capacitance values (1880 F/g) were obtained at a charge current density of 5 A/g. The samples in this experiment were tested for 2000 cycles of cycle life and still retained a reasonably good level of 76.6% of their initial capacitance value. In this study, the inverse opal structure photonic crystal substrate was used as the starting point, and then the microelectrode material for the MnS/MoS_2_/Ni-IOS supercapacitor was synthesized. Our findings show that the MnS/MoS_2_/Ni-IOS microelectrode makes a viable technical contribution to the design and fabrication of high-performance supercapacitors.

## 1. Introduction

In recent decades, the rapid development of science and technology with the steady growth in the global population has witnessed an obvious surge in fossil fuel consumption [1]. In the societal transition process, human beings have not considered the ecological protection of the natural environment, especially in developing countries. Pollution is becoming increasingly worsened, resulting in the decline of many natural resources, forcing us to address the issue of the energy crisis head-on [2]. Over the past few years, the world’s major powers have also gradually encountered the problem of insufficient energy on the supply side [3]. The delegates said that the energy crisis is not just on paper. At the 21^st^ United Nations Conference of the Parties (COP21) in 2015, a new Paris Agreement was signed, and representatives from all over the world jointly signed the document. Countries and representatives signed the agreement together and wished that the majority of the energy used worldwide could come from renewable energy sectors by 2050 [4]. 

The energy storage characteristics of supercapacitors are between secondary batteries and traditional capacitors. Compared with secondary batteries, supercapacitors can quickly store/release charges, exhibiting advantages such as high-power density, fast charge and discharge, wide operating temperatures, and long service life [5,6,7,8]. supercapacitors normally have higher energy density than traditional capacitors. Supercapacitors or electrochemical capacitors store electric charges through the unique separation of ions and charges at the microelectrode interface and form an electric double layer where the charges are separated. The electric double layer is also called electric double-layer capacitors (EDLCs), which are then divided into two categories, namely, electric double-layer capacitors and Faraday capacitors or pseudocapacitors (pseudocapacitance capacitors; PCs) according to the difference in the charge storage mechanisms [9,10,11,12]. The active material undergoes chemical reactions, mainly charge transfer storage, and pseudocapacitors tend to use transition metal oxides, sulfides, or conductive polymers as microelectrodes, and the charge storage mechanism is based on a mainly fast redox reaction but applies its electrochemical characteristics. It is not a pure capacitor nor a Faraday process. Since the energy storage characteristics are similar to an electric double-layer capacitor, a “quasi-capacitor” is a distinction [13,14,15,16,17,18]. 

This research aimed to manufacture a supercapacitor micro-cathode material with a simple process, low cost, and high specific capacitance. To ensure a higher specific surface area, adjustment of the particle size, the ratio of active substances, enhancement of the synergistic effect, etc., we envisaged using a three-dimensional (3D) ordered macroporous metal nickel structure made in our laboratory as the experimental substrate, so the structure has a high specific surface area. The advantage of this method is that the active materials such as molybdenum disulfide (MoS_2_) [19] and manganese sulfide (MnS) [20] can be more uniformly arranged and attached to the substrate. Its synergistic effect can be improved, thereby increasing the specific capacitance value of the microelectrode material. The three-dimensional ordered macroporous (3DOM) metal nickel structure is constructed by polystyrene (PS) microspheres, which are fabricated via the non-emulsification polymerization method to possess high dispersity and single particle size, and then use the electrophoretic self-assembly (EPSA) technology developed by our research team. The PS microspheres are self-assembled and deposited onto the ITO transparent conductive glass substrate to form a three-dimensional opal structure photonic crystal template. Subsequently, the pores of the opal structure are filled with metallic nickel by electrochemical deposition (ECD). Then, the PS microspheres are removed by soaking in a toluene solvent to form a metallic nickel inverse opal structure substrate. Compared with the traditional nickel foam, the metallic nickel inverse opal structure has a much higher specific surface area and can replace the bulky nickel foams as the substrate of the supercapacitor cathode materials. It is worth noting that the Mn-based cathode is also distinguished by its low toxicity, plentiful supply, exceptional safety, and environmental friendliness [21,22], and the supercapacitor synthesis starts to combine two-dimensional material such as reduced graphene oxide to increase the conductivity and surface area of the electrodes [23]. Therefore, the active material MnS/MoS_2_ was deposited into the void space of a metallic nickel inverse opal structure through an electrochemical deposition method, and the ordered porous microelectrode material MnS/MoS_2_/Ni-IOS with a high specific surface area and high specific capacitance was formed.

In this work, polystyrene (PS) photonic crystals were prepared by electrophoretic self-assembly (EPSA) as sacrificial templates and were fabricated by electrochemical deposition (ECD). A nickel inverse opal structure (Ni-IOS) was then formed. Then, the active materials of molybdenum disulfide (MoS_2_) and manganese sulfide (MnS) were sequentially deposited on the surface of the Ni-IOS test pieces. Finally, MnS/MoS_2_/Ni-IOS composite microelectrode materials were achieved, and the obtained samples were fully rinsed several times with deionized water (DI water) and anhydrous alcohol (EtOH) and dried. Furthermore, the MnS/MoS_2_/Ni-IOS composite microelectrode material was subjected to the appropriate surface analysis treatment and electrical test analysis. The optimal control of various process parameters is expected to produce MnS/MoS_2_/Ni-IOS composites to render a high specific surface area, high specific capacitance, and high cycle life. It is worth mentioning that the electrochemical deposition method was used in this experiment to deposit the desired active material on the Ni-IOS substrate, which is more straightforward than other methods in the literature.

## 2. Materials and Methods

The brands, models, and purity of the chemicals involved in the various synthesis and self-assembly methods used in this study are described as follows: styrene (CH_2_CHC_6_H_5_, purity 99%, Alfa Aesar, Haverhill, MA, USA), sodium bicarbonate (NaHCO_3_, 99%, Sigma-Aldrich, St. Louis City, MO, USA), potassium persulfate (K_2_S_2_O_8_, 99% J.T. Baker Company, Leicestershire, UK), thioacetamide (C_2_H_5_NS, 99%, Alfa Aesar, Haverhill, MA, USA), nickel chloride (NiCl2·6H_2_O, 96%, Showa Chemical Company, Tokyo, Japan), nickel sulfate (NiSO_4_·6H_2_O, 98%, Alfa Aesar, Haverhill, MA, USA), boric acid (H_3_BO_3_, 99%, Alfa Aesar, Haverhill, MA, USA), sodium hydroxide (NaOH, 97%, Showa Chemical Company, Tokyo, Japan), manganese sulfate (MnSO_4_, 99%, Thermo Corporation, Waltham, MA, USA), toluene (C_6_H_5_CH_3_, 99.78%, Jingming Chemical Company, Toufen City, Taiwan), sodium thiosulfate (Na_2_S_2_O_3_, 99%, American Fluka Company, Haverhill, MA, USA), molybdate (MoO_3_·H_2_O, 85%, Alfa Aesar, Haverhill, MA, USA), thiourea (CH_4_N_2_S, 99%, Acros Organics, Waltham, MA, USA), potassium hydroxide (KOH, 85%, Sigma-Aldrich), and ammonia (NH_4_OH, 28%, Japan Showa Company, Tokyo, Japan). 

**Figure 1:**
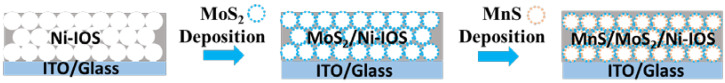
Schematic diagram of the experimental setup for the electrophoretic self-assembly (EPSA) method.

In this work, polystyrene (PS) photonic crystals were prepared by electrophoretic self-assembly (EPSA) as sacrificial templates and were fabricated by electrochemical deposition (ECD). A nickel inverse opal structure (Ni-IOS) was then formed. Then, the active materials of molybdenum disulfide (MoS_2_) and manganese sulfide (MnS) were sequentially deposited on the surface of the Ni-IOS test pieces. Finally, MnS/MoS_2_/Ni-IOS composite microelectrode materials were achieved, and the obtained samples were fully rinsed several times with deionized water (DI water) and anhydrous alcohol (EtOH) and dried. Furthermore, the MnS/MoS_2_/Ni-IOS composite microelectrode material was subjected to the appropriate surface analysis treatment and electrical test analysis, as shown in Figure 1. The optimal control of various process parameters is expected to produce MnS/MoS_2_/Ni-IOS composites to render a high specific surface area, high specific capacitance, and high cycle life. It is worth mentioning that the electrochemical deposition method was used in this experiment to deposit the desired active material on the Ni-IOS substrate, which is more straightforward than other methods in the literature.

### 2.1. Electrophoretic Self-Assembly (EPSA) 

When starting EPSA, we will first prepare the suspension and mix our self-synthesized single-size PS microspheres (average particle size 530 ± 20 nm) with a solvent with a water–alcohol ratio (DI water:ethanol = 3:7) evenly mixed and shaken with ultrasonic agitation for at least 30 min, and finally prepared into a 1 wt% electrophoresis (EP) suspension with uniform fluidity. 

**Figure 2:**
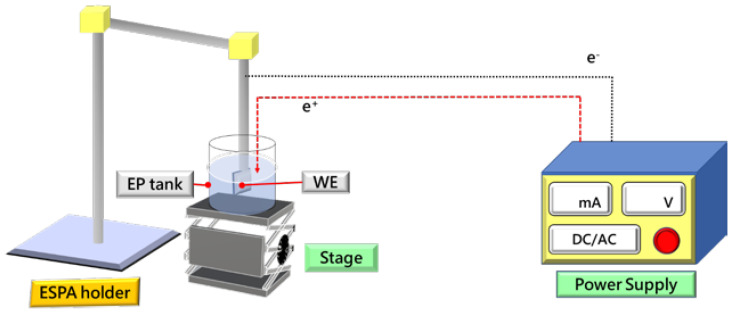
Schematic diagram of the experimental setup for the EPSA method.

In the process of EPSA, we used the transparent conductive ITO/glass material as the working electrode (WE) and the pure platinum metal sheet as the opposite electrode (CE). The constant voltage mode sets various electric field intensities and coating times. The single and dispersed PS microspheres are self-assembled to form a coating layer to make a three-dimensional (3D) photonic crystal template. Subsequently, the PS microsphere template prepared by our unique EPSA coating method served as the follow-up fabrication of a metallic nickel inverse opal structure. Although the PS microsphere photonic crystal template acts as a sacrificial template because its structural compactness will significantly affect the formation of the nickel inverse opal structure, this section focuses on the effects of various EPSA parameters on the PS template. Figure 2 illustrates our self-designed electrophoretic self-assembly (EPSA) experimental setup.

### 2.2. Electrochemical Deposition (ECD)

Electrochemical deposition experiments are mainly carried out using a three-electrode cell, whose reaction device includes [1] a counter electrode (CE) whose primary function is to pass current to form a loop and aid the deposition of the desired deposit on the working electrode. The counter electrode is generally more extensive in area than the working electrode to reduce the current density on the opposing electrode and can improve the current of the working electrode itself and distribute the current more evenly [24]; the reference electrode (RE) is used as a three-electrode electrochemical deposition in the chemical deposition tank. Its function is because the ECD process needs to have a stable potential and can stabilize the potential difference between the opposite electrode and the working electrode. It is not easy to produce significant potential changes, and the reaction potential is still maintained at a particular value [25]; the working electrode (working electrode; WE) is the substrate to be deposited, and the WE is the cathode of the electrochemical deposition reaction. Note that the ions in the electrolyte will be reduced and deposited on the surface of the working electrode.

In general, the electroplating method belongs to the scope of electrochemical deposition technology, which gives rise to the surface of the working electrode to deposit a pure metal film. Since depositing nickel metal is relatively stable, the general practice can temporarily ignore the setting of the reference electrode, so we adopted the two-pole method. The device undergoes an electrochemical deposition process, and we will use this design to fabricate a nickel inverse opal structure. The bath formula required for nickel electroplating includes NiSO_4_·6H_2_O (0.84 M), NiCl_2_·6H_2_O (0.23 M), and H_3_BO_3_ (0.29 M), which are prepared by adding a small number of low concentrations of H_2_SO_4_ or NaOH. The pH of the plating solution itself can be adjusted. According to the mixed composition formula of the chemicals, as mentioned earlier, after stirring for 24 h, the preparation steps of the required plating solution are completed. First, the fabricated 3D PS photonic crystal template was immersed in the electroplating solution, and the electroplating process was performed using the constant current mode of an electrochemical workstation. After the preparation of the test piece after electroplating, we carefully rinsed the sample with deionized water and alcohol several times, and then put in an oven (60 °C) to dry for at least 2 h, then immersed the prepared sample in toluene for several days to remove the polymeric substance of the PS microspheres. The microspheres were removed and rinsed thoroughly with DI water and alcohol for several runs to complete the metallic nickel inverse opal structure preparation. Next, we used the ECD method to prepare molybdenum disulfide on the surface of the nickel inverse opal structure.

First, molybdic acid (H_2_MoO_4_, 0.1 M) and sodium thiosulfate (Na_2_S_2_O_3_, 0.2 M) were dissolved in an ammonia water [NH_3(aq)_ or NH_4_OH_(aq)_] solvent to establish a molybdenum disulfide electroplating solution. The working temperature was controlled at 5 °C, and the electrochemical deposition was performed in constant current mode. At the same time, different constant currents and deposition times were used to find the best parameters. After the electrodeposition step, we used DI water and alcohol to clean the MoS_2_/Ni-IOS several times, and put the specimen into the oven until the sample was dried. Then, the sample was subjected to a heat treatment step (temperature 300 °C/1 h), wherein the heating rate was 2 °C/min, and the final sample passed through the surface analysis test (SEM, XRD, XPS, and TEM), thereby the optimal electrodeposition parameters were confirmed. Next, we used the ECD method to prepare manganese sulfide on the surface of the molybdenum disulfide/nickel inverse opal structure. First, two chemicals, manganese sulfate (MnSO_4_, 5 mM) and thiourea (CH_4_N_2_S, 0.75 M), were dissolved in deionized water to form a molybdenum sulfide electroplating solution. A three-pole electrode was used, in which foamed nickel was the working electrode (WE), and a pure metal platinum (Pt) sheet was the opposite electrode (CE). Then, Ag/AgCl was used as the reference electrode (RE), and the constant current mode was applied through the electrochemical workstation, and the electrodeposition step was performed. At the same time, different constant currents and deposition times were used to find the most optimum parameters and finally complete the MnS/MoS_2_/Ni-IOS sample. After the electrodeposition step was completed, we used DI water and alcohol to wash the test piece several times and then put into the oven to dry the sample. Through various analytical means such as SEM, cyclic voltammetry (CV), the galvanostatic charge–discharge (GCD) test, and cycle life test for the final sample, the optimal electrodeposition parameters were confirmed and evaluated.

## 3. Results and Discussion

### 3.1. Microstructure Analysis of Manganese Sulfide/Molybdenum Disulfide

**Figure 3:**
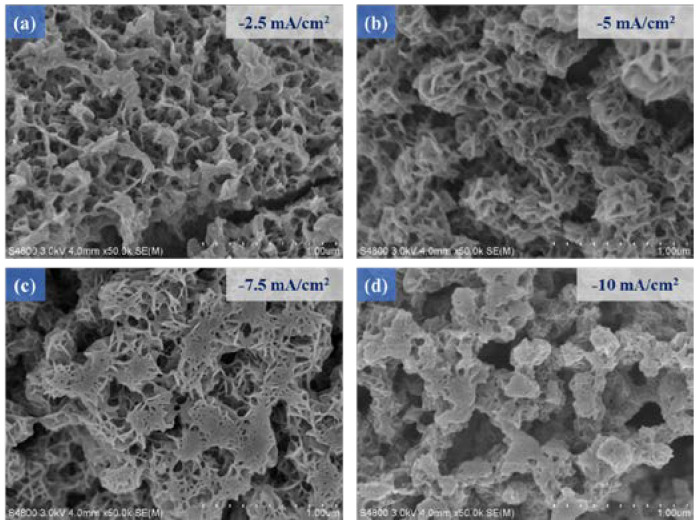
The MnS/MoS_2_ thin films were prepared by electrodeposition with the current densities of (**a**) −2.5 mA/cm^2^, (**b**) −5 mA/cm^2^, (**c**) −7.5 mA/cm^2^, (**d**) −10 mA/cm^2^, respectively.

The SEM micrographs of Figure 3a–d respectively show that the MnS thin films prepared under different current densities (−2.5 mA/cm^2^–10 mA/cm^2^) were displayed on the surface of the MoS_2_/ITO glass template microstructure. When the current density increased, the phenomenon of agglomeration occurred between the MnS films. A higher current density will improve the bonding strength between the MnS films. Therefore, when we subsequently conducted the electrochemical deposition of the MnS films, we decided to choose the conditions of high current density to prepare these MnS thin films (current density −10 mA/cm^2^). In addition, longer deposition time conditions will give rise to the generation of a MnS flower-like structure showing a micro-spherical patterned structure, so the deposition time was finally selected as 10 min to achieve the optimal MnS/MoS_2_ composite material. 

### 3.2. Preparation of Manganese Sulfide/Molybdenum Disulfide/Ni-IOS by Electrochemical Deposition

#### 3.2.1. Preparation of MoS_2_/Ni-IOS with Different Deposition Times

**Figure 4:**
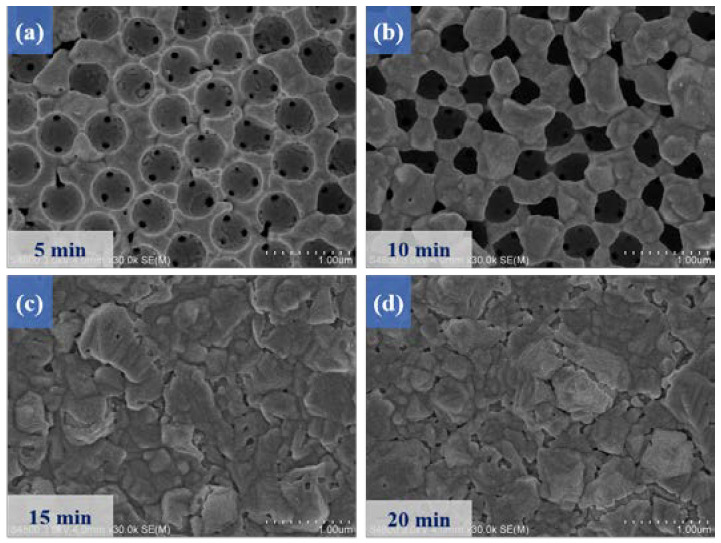
Top-view of SEM images of MoS_2_/Ni-IOS microelectrode samples fabricated with different electrodeposition times, where the electrodeposition parameter was −10 mA/cm^2^, and the deposition times were (**a**) 5 min, (**b**) 10 min, (**c**) 15 min, (**d**) 20 min.

A constant current density of −10 mA/cm^2^ was applied to deposit MoS_2_ thin films on the surface of a nickel-based inverse opal structure, and we further explored the electrochemical deposition time of MoS_2_. Figure 4 shows the microstructure images of MoS_2_ deposited on nickel inverse opal structures at different deposition times. Figure 4a shows that when the deposition time was 5 min, the nickel inverse opal structure had not been entirely covered by the MoS_2_ film, and the existence of the nickel inverse opal structure could also be seen.

When the deposition time was increased to 10 min, it was found in Figure 4b that the MoS_2_ film covered the nickel inverse opal structure, and even some holes or voids appeared. Taking advantage of supercapacitors also benefits the deposition of subsequent MnS films on the MoS_2_/Ni-IOS substrates. Under the premise of maintaining the nickel inverse opal structure, we tried to avoid the deposition of active materials to block the Ni-IOS pores, and thus obtain the advantageous feature of a high specific surface area.

**Figure 5:**
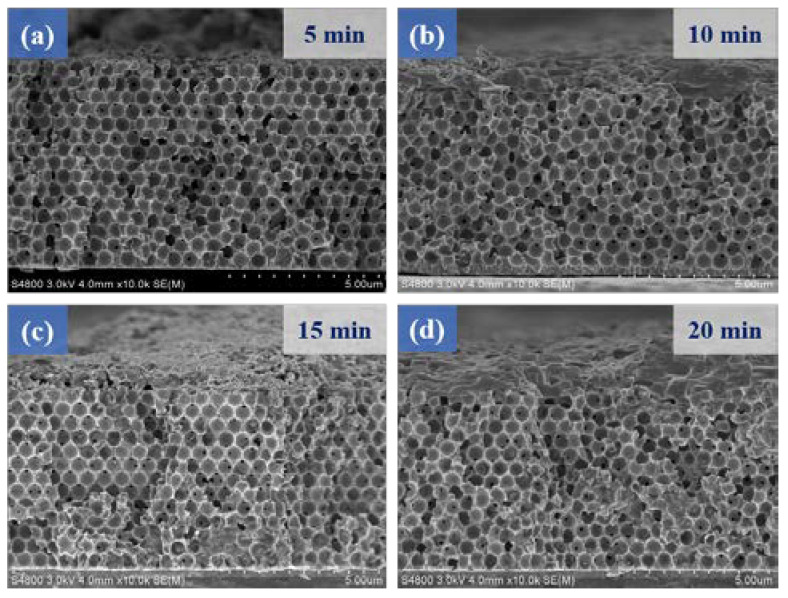
Cross-sectional SEM images of the MoS_2_/Ni-IOS microelectrode samples fabricated with different electrodeposition times, with a −10 mA/cm^2^ electrodeposition parameter, and the deposition times were (**a**) 5 min, (**b**) 10 min, (**c**) 15 min, (**d**) 20 min.

Figure 5 shows the cross-sectional microstructures of MoS_2_/Ni-IOS substrates with different deposition time parameters. The experimental results in Figure 5a show that the structure of the nickel inverse opal was complete and unmodified. We speculate that the reason is that the deposition time was too short, resulting in incomplete deposition of the MoS_2_ film. When we prolonged the deposition time to 10 min, Figure 5b depicts a MoS_2_ active material that can smoothly enter and fill the hole space at the bottom of Ni-IOS; as shown in Figure 5, the inside of the sample was uniformly filled with a MoS_2_ thin film. Finally, when we continuously increased the deposition time to both 15 min and 20 min, although Figure 5c,d shows that they filled with Ni-IOS more completely, the surface was filled and saturated at this time. The inability to deposit MnS thin films in the inner pores was not only hindered by the composite synthesis step, but also failed to exhibit the advantages of the porous structure of the Ni-IOS substrate. MoS_2_ HRTEM image, EDS analysis, TEM SAED analysis, and XPS elemental composition analysis are shown in Figure S1, Figure S2, and Figure S3, respectively. 

#### 3.2.2. Preparation of Manganese Sulfide/Molybdenum Disulfide/Ni-IOS with Different Deposition Current Densities

**Figure 6:**
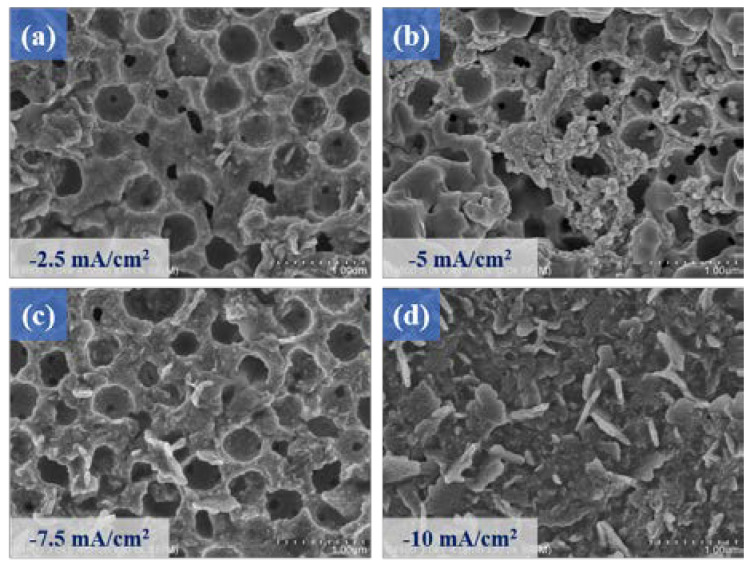
Top-view SEM images of the MnS/MoS_2_/Ni-IOS microelectrode samples fabricated with different electrodeposition densities, where the 10 min electrodeposition parameters of MnS were selected, and the current densities were (**a**) −2.5 mA/cm^2^, (**b**) −5 mA/cm^2^, (**c**) −7.5 mA/cm^2^, and (**d**) −10 mA/cm^2^.

From the previous results of MoS_2_/Ni-IOS, too long a deposition time will lead to the densification of the structure and cause the subsequent MnS film to not achieve good spatial growth. Hence, we used −10 mA/cm^2^, 10 min as the electroplating MoS_2_ parameters. Subsequent experimental work will discover the effect of the current density of MnS film deposition at a fixed deposition time (10 min). Figure 6 shows the microstructure image of MnS deposited on the MoS_2_/Ni-IOS substrate at different current densities. When the deposition density was −2.5 mA/cm^2^ as shown in Figure 6a, it was observed that the MnS film was uniformly deposited on the MoS_2_/Ni-IOS substrate. Since many active reaction sites are involved in facilitating the exchange of electrons, we speculate that the composite material’s specific capacitance value will escalate on the MoS_2_/Ni-IOS substrate. Subsequently, the CV results shown in figure in Section 3.3 and discussed below will match and support this argument. With an increase in the current density of the electrodeposition process, Figure 6b–d shows that the original MoS_2_/Ni-IOS substrate was filled with the MnS coating (MnS HRTEM EDS, XRD, XPS, HRTEM SAED analysis are shown in Figure S4, Figure S5 and Figure S6; MnS/MoS_2_ composition by TEM EDS analysis is shown in Figure S7). Still, when it was filled with the coverage of active material, it could not reveal the porous nature of MoS_2_/Ni-IOS and its advantages of multiple reaction sites. In addition, we observed that when higher current densities were used in the electrodeposition process, the densification of the MnS films was also perfect.

**Figure 7:**
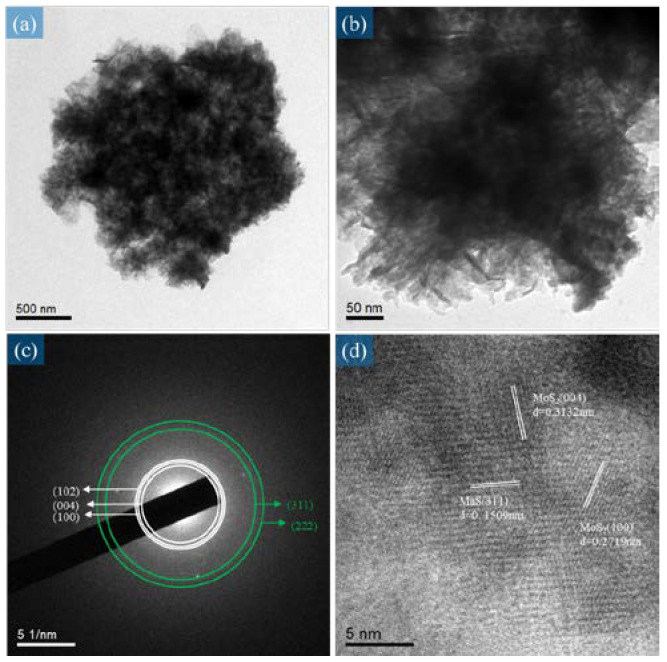
(**a**,**b**) TEM microscopic image analysis image of the MnS/MoS_2_ composite film at different magnifications, (**c**) TEM selective diffraction image of the MnS/MoS_2_ composite material, (**d**) TEM high solution image showing the lattice fringe of MnS/MoS_2_.

As depicted in Figure 7a, we know that the MnS/MoS_2_ thin film we produced presented a multi-layer structure, where MnS comes up with a sheet structure and MoS_2_ with a layer structure, which can be corroborated by SEM microscopic images given separately below. From the comparison of the selected area diffraction pattern (SAED) in Figure 7c with the XRD pattern (as shown in Figure S8), we found the diffraction peaks (102), (004), and (100) of MoS_2_ and the diffraction peaks (311) and (222) of MnS. Obviously, this is consistent with the XRD data above-mentioned. It was also confirmed that the MnS/MoS_2_ composite prepared by the electrodeposition route is rather suitable for the design of supercapacitor samples.

**Figure 8:**
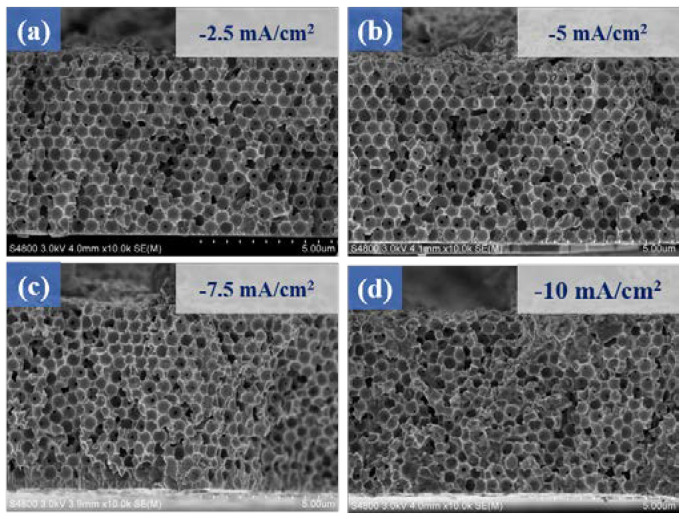
Cross-sectional micrographs of the MnS/MoS_2_/Ni−IOS microelectrode samples fabricated with different electrodeposition densities. The current densities were (**a**) −2.5 mA/cm^2^, (**b**) −5 mA/cm^2^ using the 10 min electrodeposition parameters of MnS, (**c**) −7.5 mA/cm^2^, and (**d**) −10 mA/cm^2^, respectively.

Figure 8 shows the cross-sectional microstructure images of MnS deposited on MoS_2_/Ni-IOS under different current densities (Figure 8a). When using a low current density to deposit the MnS film, it was evident that Ni-IOS maintained a fine porous structure (BET data is given in Figure S9) to facilitate the entry of electrolytes into the interior for the proceeding ion exchange, which is quite crucial for electrochemical performance such as specific capacitance value. Figure 8b–d shows that when the deposition current density increased, the internal pores gradually filled up, which is undoubtedly a significant disadvantage for exchanging or storing charges between ions. Finally, we speculate that the optimal electrodeposited MnS film parameter was −2.5 mA/cm^2^ and a deposition time of 10 min.

### 3.3. Cyclic Voltammetry of Manganese Sulfide/Molybdenum Disulfide/Ni-IOS

**Figure 9:**
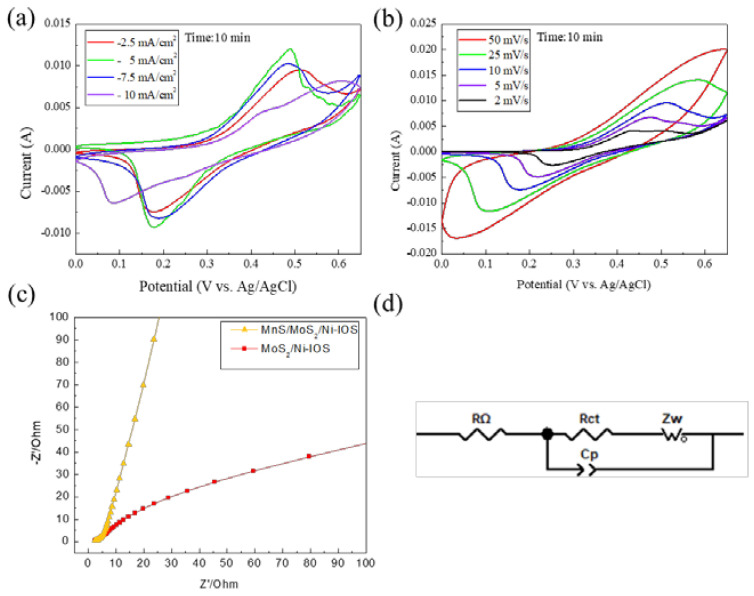
(**a**) CV test curves of the MnS/MoS_2_/Ni-IOS microelectrode samples with different electrodeposition current densities for a 10 min deposition time, where the scan rate was 10 mV/s, and the current densities were −2.5 mA/cm^2^, −5 mA/cm^2^, −7.5 mA/cm^2^, and −10 mA/cm^2^, respectively. (**b**) CV curves of the MnS/MoS_2_/Ni-IOS microelectrode samples at different scan rates, using the −2.5 mA/cm^2^ and 10 min electrodeposition parameters, the scan rates were 50 mV/s, 25 mV/s, 10 mV/s, 5 mV/s, and 2 mV/s, respectively. (**c**) Nyquist plots of the MoS_2_/Ni-IOS and MnS/MoS_2_/Ni−IOS microelectrode samples, and (**d**) the equivalent circuit used for fitting the impedance spectra of the MnS/MoS_2_/Ni-IOS microelectrode samples.

From Figure 9a, the constant current density of −10 mA/cm^2^ and deposition time of 10 min were the parameters used for the MoS_2_ micro-electrodeposition process and matched with different current densities and constant deposition time (−2.5 to −10 mA/cm^2^ and 10 min) to deposit a MnS thin film. Finally, a composite microelectrode material MnS/MoS_2_/Ni-IOS was prepared, and then the test piece was tested by cyclic voltammetry with a scan rate of 10 mV/s. It can be seen from Figure 9a that due to the deposition current density of −2.5 mA/cm^2^, the active material seemed lighter, and it can be seen from Figure 6 and Figure 8 that there were more nickel inverse opal structure holes. Notably, when the current density of the electrochemical deposition was −2.5 mA/cm^2^, more active sites facilitated the exchange effect of surface ions during the CV test. The final results of better specific capacitance in achieving the purpose of charge storage corresponded to the CV test curves in Figure 9a. It can be concluded that the optimal electrodeposition parameters of the microelectrode material sample of the MnS/MoS_2_/Ni-IOS thin film electrodeposited by us were the deposition current density of −2.5 mA/cm^2^, the deposition time of 10 min, and the specific capacitance value of the sample obtained was 1398 F/g as shown in Table 1.

Figure 9b shows the cyclic voltammograms of the MnS/MoS_2_/Ni-IOS thin film microelectrode material samples with optimal micro-electrodeposition parameters at different scan rates. At high scan rates, the area of the CV curve was more extensive, but this did not allow the microelectrode material to store more electrical charges. With the higher scan rate, the current density of the sample will escalate, causing the oxidation peak to shift to the positive potential direction and the reduction peak to shift to the negative potential direction. This situation is similar to Ghasemi’s work, and their CV shapes were slightly distorted at high scan rates because the entire active surface of the electrode did not fully participate in electrochemical processes at high scan rates. Furthermore, as the scan rate increased, the area of the CV curves accordingly increased, which does not imply more ion storage [25,26]. In brief, the experimental results were obtained due to the dual effects of simultaneous phase change and the polarization reaction of the samples under high scan rate conditions.

The phase change behavior involved here is the dissociation of the active species during the electrochemical reaction, resulting in an irreversible reaction during the redox process. The polarization reaction is due to the irreversible redox process accompanying the phase change of the active material, which further shifts the redox peaks. Therefore, as the scan rate is increased, the active material must be able to induce a redox reaction under the over potential condition, but selecting a higher scan rate condition will obtain a more significant charge transfer resistance between the microelectrodes, which can be seen from another angle, where the Faraday redox reaction of the composite material manifests strong irreversibility. We predict that if the potential range is widened, redox peaks at high scan rates will inevitably be encountered [17,18].

The Nyquist plots of MoS_2_/Ni-IOS and MnS/MoS2/Ni-IOS microelectrodes, spanning from 100 kHz to 0.01 kHz, are shown in Figure 9c. The internal resistance (R in Ω) of the material is shown by the intercept of the X-real axis (Z’). The redox reaction and double layer reaction on the surface of the materials cause the size of the semicircle in the high-frequency region of curves on behalf of the charge-transferring impedance (R_ct_). Meanwhile, in the low-frequency region, the slope of the line is strongly connected to the diffusion impedance (Warburg impedance, Z_w_) from the electrolyte ions, with a larger slope representing a shorter diffusion distance for electrolyte ions, which leads to reduced diffusion resistance. Furthermore, Figure 9c illustrates that the composite lacks an apparent semicircle, implying a low charge-transferring impedance. This indicates that the MoS_2_/Ni-IOS and MnS/MoS_2_/Ni-IOS microelectrodes have a rapid charge transfer capacitance and tight contact between the electrochemically active material and the current collector. The MnS/MoS_2_/Ni-IOS microelectrode has a bigger vertical slope than the MoS_2_/Ni-IOS sample in the low-frequency range. Our findings show that the electrolyte had the superior permeability of the MnS/MoS_2_/Ni-IOS microelectrodes, and the electrolyte possessed less diffusional resistance in the MnS/MoS_2_/Ni-IOS microelectrodes. As a consequence of the introduction of MnS, the MnS/MoS_2_/Ni-IOS microelectrode had greater advantages as the supercapacitor electrode material.

### 3.4. Charge–Discharge and Cycle Life Test of Manganese Sulfide/Molybdenum Disulfide/Ni-IOS Microelectrodes

**Figure 10:**
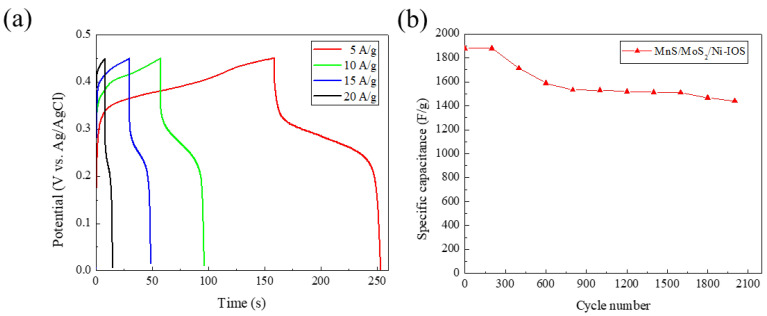
(**a**) The galvanostatic charge–discharge test (GCD) curves of the MnS/MoS_2_/Ni-IOS microelectrode samples with different charge–discharge currents, where the potential range was 0~0.45 V, and the current densities were 5 A/g, 10 A/g, 15 A/g, 20 A/g, respectively. (**b**) The cycle life test results of the MnS/MoS_2_/Ni-IOS microelectrode material prepared by using a current density of 5 A/g and a round number of 2000 cycles.

Figure 10a illustrates the MnS/MoS_2_/Ni-IOS microelectrode material sample with the best parameters (electrodeposition current density −2.5 mA/cm^2^ and electrodeposition time 10 min) under a specific potential range (0.0~0.45 V). With various current densities (5~20 A/g), the graph of the galvanostatic charge/discharge cycle (GCD) test of the test piece can be obtained. The GCD measurement was carried out to evaluate the material’s supercapacitor performance (specific capacitance value, Coulomb efficiency, and rate efficiency) under kinetic energy [26]. The following formula (1) is used to calculate the specific capacitance measured by GCD at a current density of 5 A/g value [27]:

(1) $$C_{specific}=\frac{2\times I\times \int Vdt}{\Delta V^{2}\times m}$$

where $C_{specific}\;$ is the specific capacitance value (F/g); I is the discharge current (A); ∫Vdt is the area of the discharge curve (A); ΔV is the potential range; and m is the active material weight (g).

The specific capacitance data collected for the MnS/MoS_2_/Ni-IOS microelectrode samples under various current density test conditions are shown in Figure 10a. The experimental results of the obtained MnS/MoS_2_/Ni-IOS microelectrodes show that during the charging and discharging process, the material could still achieve an excellent specific capacitance value (1880.5 F/g) under the condition of a current density of 5 A/g.

Generally, the rate capability refers to the excellent stability of the sample under high current density. Admittedly, the rate capability of the sample we prepared may need to be improved for future work. When the sample underwent a higher charge–discharge current density of 10 A/g, the specific capacitance value remained at 1503.4 F/g, only maintaining its former ratio, equivalently the capacitance value of the 79.9% level. We can observe from Figure 10a that the magnification capability of this sample is not yet ideal. Due to the dual effects of phase change and the polarization reaction of the MnS/MoS_2_/Ni-IOS active material corresponding to Figure 9b operating under high scan rate conditions, the conceivable reasons are given as follows: (1) a raise in the charging platform, and (2) too significant an IR drop. When the charging platform is lifted up, the sample cannot be charged suitably to a broader potential range under high current density situation, so the specific capacitance value is reduced. We speculate that the cause of the IR drop is due to the excessive charge transfer resistance (R_ct_) between the MnS and MoS_2_ films, which results in inconsistent charge–discharge platforms and thus weakens the overall electrochemical performance.

The previous experimental results confirm that the high specific surface area metal Ni-IOS substrate produced in this experiment can ideally replace the traditional nickel foam substrate as a better working microelectrode material, and it certainly ensures that the overall performance of the specific capacitance value of the supercapacitor is improved. In other words, under a current density of 5 A/g, the MnS/MoS_2_/Ni-IOS microelectrode material fabricated in our work delivered a satisfactory performance of specific capacitance (1880.5 F/g) as shown in Table 2.

Figure 10b illustrates the MnS/MoS_2_/Ni-IOS microelectrode material prepared by the electrochemical deposition method under the condition of a current density 5 A/g, 2000 times of charge, and discharge cycle life test. After a trial of 2000 cycles, the prepared microelectrode material decreased from the original specific capacitance value of 1880 F/g to 1441.2 F/g, and the cycle retention rate was 76.6%. It is postulated that the main reason is the improvement in the potential platform and the charge transfer, even through multiple cycles. Due to the relatively high resistance, an increase in the potential platform caused the actual specific capacitance value to be much higher than the measured one. In general, the charge transfer resistance (R_ct_) shows that the outcome of specific capacitance will be diminished during the ion exchange process, resulting in a decrease in the specific capacitance value after each cycle. This is attributed to the R_ct_ between the composites MnS/MoS_2_ and the phase change of the active species, profoundly resulting in a polarization reaction. Therefore, the cycle retention rate of 76.6% is evidence to demonstrate that the MnS/MoS_2_/Ni-IOS microelectrode material prepared by us had extremely high cycle stability.

## 4. Conclusions

In this work, we prepared MoS_2_ and MnS deposited onto a nickel foam and inverse opal structure by electrochemical deposition to fabricate supercapacitor microelectrodes and analyzed them. The processes of our research themes include the following: (1) Electrophoretic self-assembly (EPSA) for PS microspheres self-assembled and coated on an ITO glass substrate to form a three-dimensional photonic crystal structure built by PS microspheres; (2) electrochemical deposition was used to fabricate photonic crystals with metal nickel in an inverse opal structure; (3) the composition of MoS_2_ and MnS thin films was analyzed; (4) fabrication of MoS_2_/Ni-foam, MnS/Ni-foam, and MnS/MoS_2_/Ni-IOS structured microelectrodes by the electrochemical deposition method; and (5) microstructure analysis and electrochemical analysis of the MnS/MoS_2_/Ni-IOS test. The Nyquist curves of the MoS_2_/Ni-IOS and MnS/MoS_2_/Ni-IOS structured microelectrodes and the equivalent circuit showed low charge-transferring impedance with a relatively shorter diffusion distance for electrolyte ions. The MnS/MoS_2_/Ni-IOS microelectrodes exhibited rapid charge transfer capacitance, which is because of the tight contact between the electrochemically active material and the current collector. The final sample achieved an excellent specific capacitance value (1880 F/g) at a charge–discharge current density of 5 A/g. After 2000 cycles of cycle life testing, the sample in this experiment still held 76.6% of the initial capacitance value.

## Figures and Tables

**Table 1:** The specific capacitance value of the sample by CV with a scan rate of 10 mV/s and 10 min deposition time.

Deposited current density (mA/cm^2^)	−2.5	−5	−7.5	−10
Specific capacitance (F/g)	1398	830	438	347

**Table 2:** The specific capacitance value of the sample by CV with a scan rate of 10 mV/s and 10 min deposition time.

Substrate	Fabrication Methods	Testing Parameter	Specific Capacitance Value(F/g)	Materials	References
Al_2_O_3_	Hydrothermal	1 A/g	201	C/MoS_2_	[28]
steel	Hydrothermal	5 A/g	452.7	MoS_2_ /MWCNTs	[29]
steel	Hydrothermal	1 A/g	570	MoS_2_ /rGO/PANI	[30]
Nickel Foam	Hydrothermal	5 mV/s	742	1T/2H MoS_2_	[31]
Nickel Foam	Hydrothermal	10 mV/s	289	Y_2_Zr_2_O_7_/MnS	[32]
Nickel Foam	Hydrothermal	1 A/g	627	MnS/Co_3_S_4_	[33]
Nickel Foam	Hydrothermal	0.5 A/g	300	MnS/NPC/ rGO	[34]
Nickel Foam	Hydrothermal	2 A/g	1248	MnS/rGO	[35]
Ni-IOS	Electrodeposition	5 A/g	1880	MnS/MoS_2_	This work

## Data Availability

The datasets generated during and analyzed during the current study are available from the corresponding author upon reasonable request.

## Supplementary Materials

The following supporting information can be downloaded at: https://www.mdpi.com/article/10.3390/mi14020361/s1, Figure S1: TEM energy dispersive analysis (EDS) image of MoS_2_ thin film and elemental mapping analysis.; Figure S2: (a,b) are the HRTEM image of MoS_2_ at different magnifications, (c) is the TEM SAED image MoS_2_, (d) is the HRTEM image shows the lattice fringe of MoS_2_.; Figure S3: (a) XRD diffraction pattern of MoS_2_ film coated on ITO/glass, (b) XPS spectrum of Mo 3d orbitals from XPS electronic band analysis of MoS_2_ thin films, (c) XPS spectrum of S 2p orbitals from XPS electronic band analysis of MoS_2_ thin films.; Figure S4: TEM energy dispersive analysis (EDS) image of MnS thin film and elemental mapping analysis. Figure S5: (a) XRD diffraction pattern of MnS film coated on ITO/glass, (b) XPS spectrum of Mn 2p orbitals from XPS electronic band analysis of MnS thin films, (c) XPS spectrum of S 2p orbitals from XPS electronic band analysis of MnS thin films; Figure S6: (a) is the HRTEM microscopic image analysis image of MnS thin film, (b) is the TEM selected area diffraction image of MnS, (c) is the lattice fringe image of MnS thin film; Figure S7: TEM energy dispersive analysis (EDS) image of the MnS/MoS_2_ composite; Figure S8: XRD diffraction patterns of MnS/MoS_2_ composite thin films; Figure S9: The BET adsorption curve of MnS/ MoS_2_/Ni-IOS composite thin films.
