# Supplementary material for: Electrochemically Deposited MoS2 and MnS Multilayers on Nickel Substrates in Inverse Opal Structure as Supercapacitor Microelectrodes
Source: Micromachines (Basel). 2023 Jan 31;14(2):361. doi: 10.3390/mi14020361 (PMC9961811; doi:10.3390/mi14020361)
Supplement: Supplementary file 1 [file micromachines-14-00361-s001.zip › micromachines-2077525-supplementary.pdf]

Supplementary Information

Electrochemically Deposited MoS_2 and MnS Multilayers on Nickel Substrates in Inverse Opal Structure as Supercapacitor Microelectrodes

Sheng-Kuei Chiu[1], Po-Yan Chen[1] and Rong-Fuh Louh[1,*]

Department of Materials Science and Engineering, Feng Chia University, Taichung, Taiwan

*Corresponding authors. E-mail addresses: rflouh@fcu.edu.tw

1. The MnS/MoS_2/Ni-IOS microelectrodes samples composition and structure analysis

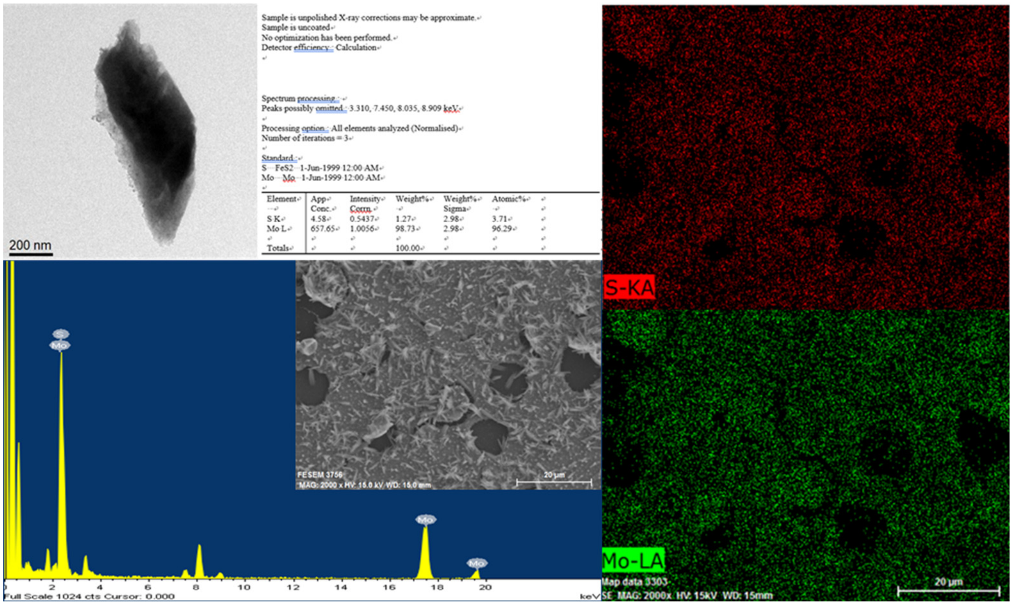

Figure S1. TEM energy dispersive analysis (EDS) image of MoS_2 thin film and elemental mapping analysis.

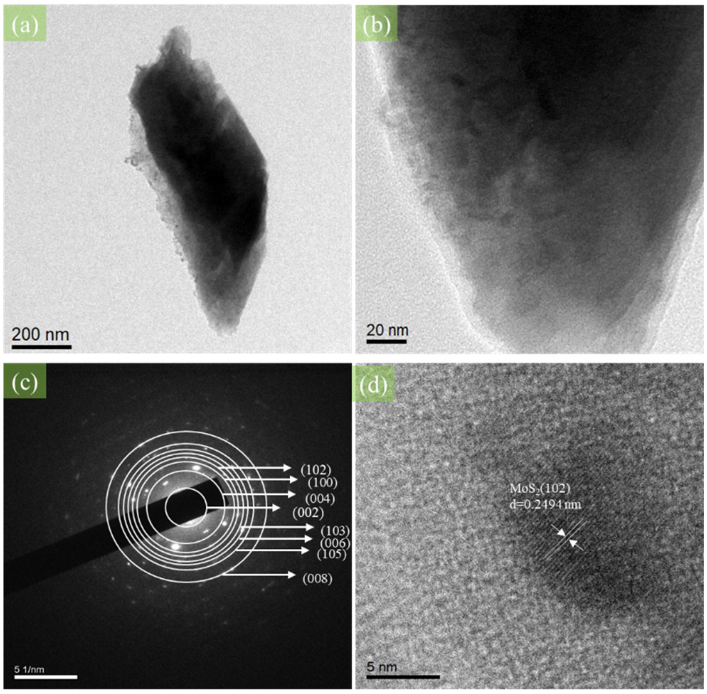

Figure S2. (a)(b) are the HRTEM image of MoS_2 at different magnifications, (c) is the TEM

SAED image MoS_2, (d) is the HRTEM image shows the lattice fringe of MoS_2.

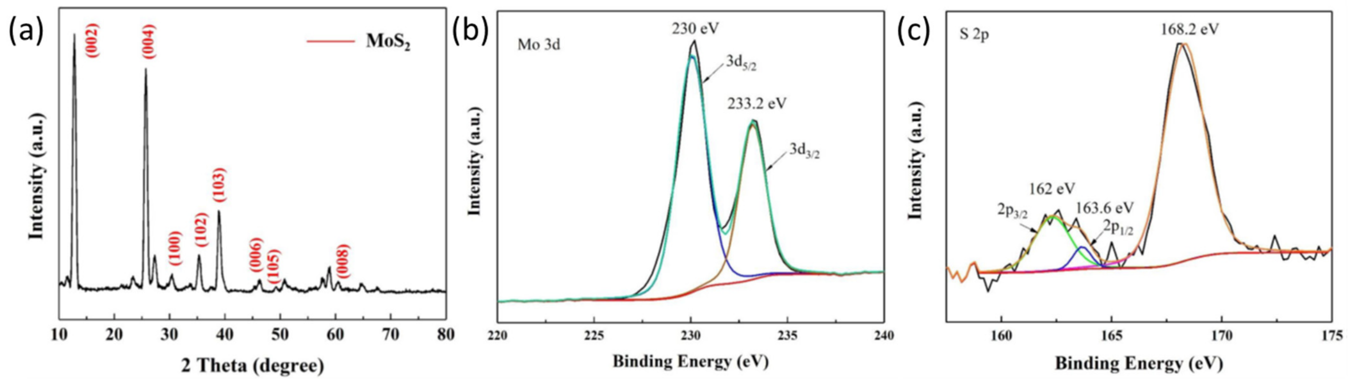

Figure S3. (a) XRD diffraction pattern of MoS_2 film coated on ITO/glass, (b) XPS spectrum of Mo 3d orbitals from XPS electronic band analysis of MoS_2 thin films, (c) XPS spectrum of S 2p orbitals from XPS electronic band analysis of MoS_2 thin films.

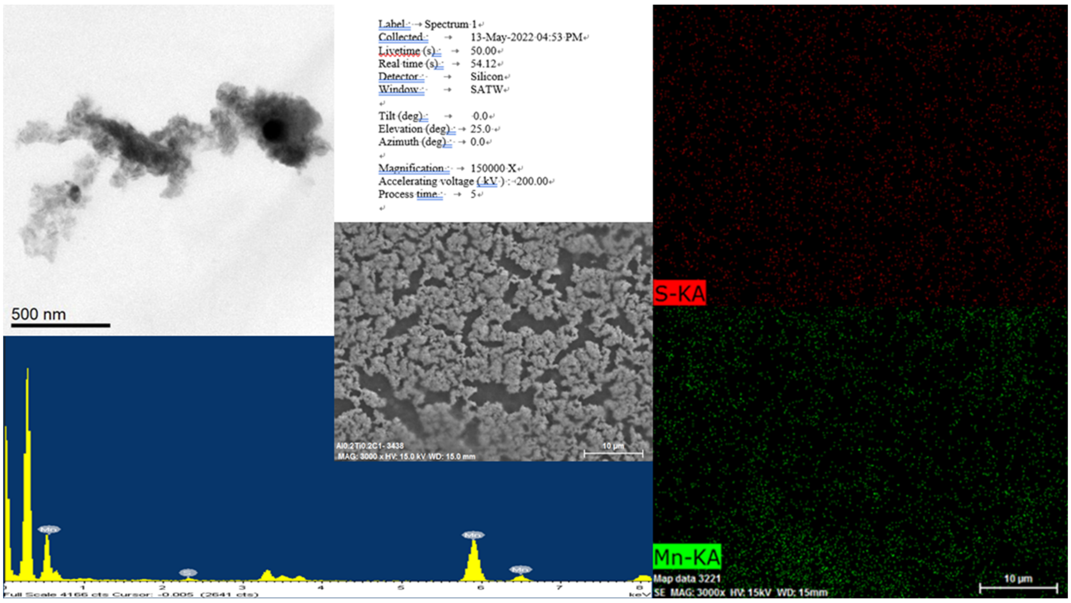

Figure S4. TEM energy dispersive analysis (EDS) image of MnS thin film and elemental mapping analysis.

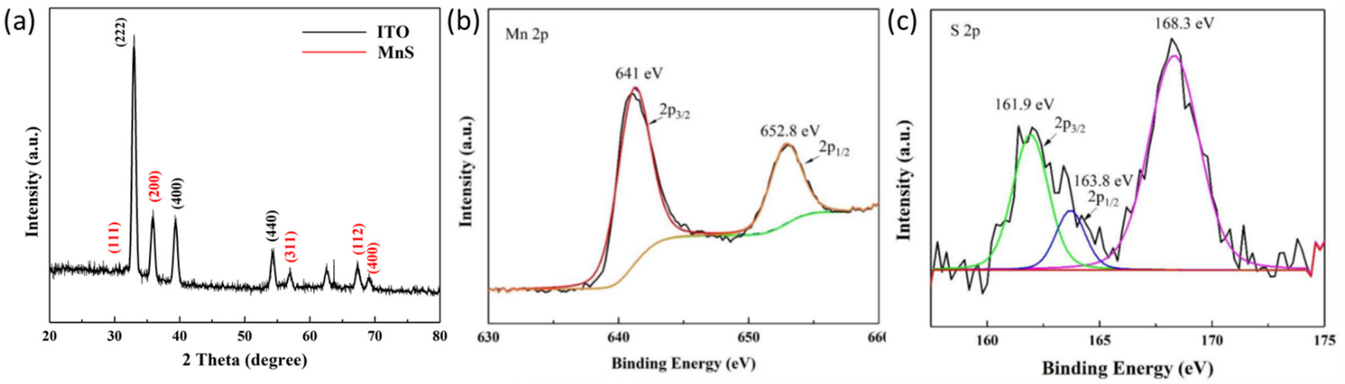

Figure S5. (a) XRD diffraction pattern of MnS film coated on ITO/glass, (b) XPS spectrum of Mn 2p orbitals from XPS electronic band analysis of MnS thin films, (c) XPS spectrum of S 2p orbitals from XPS electronic band analysis of MnS thin films.

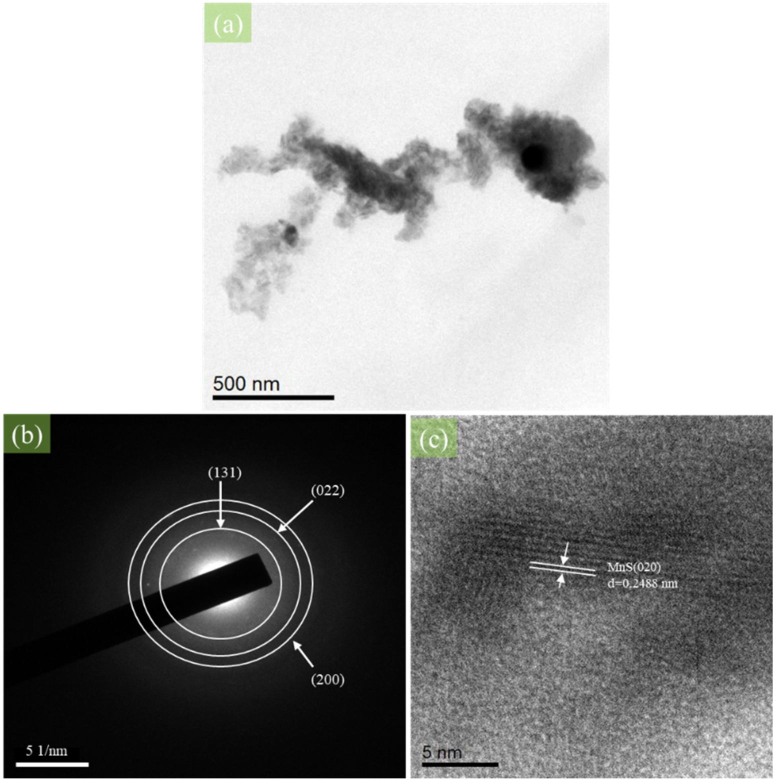

Figure S6. (a) is the HRTEM microscopic image analysis image of MnS thin film, (b) is the TEM selected area diffraction image of MnS, (c) is the lattice fringe image of MnS thin film

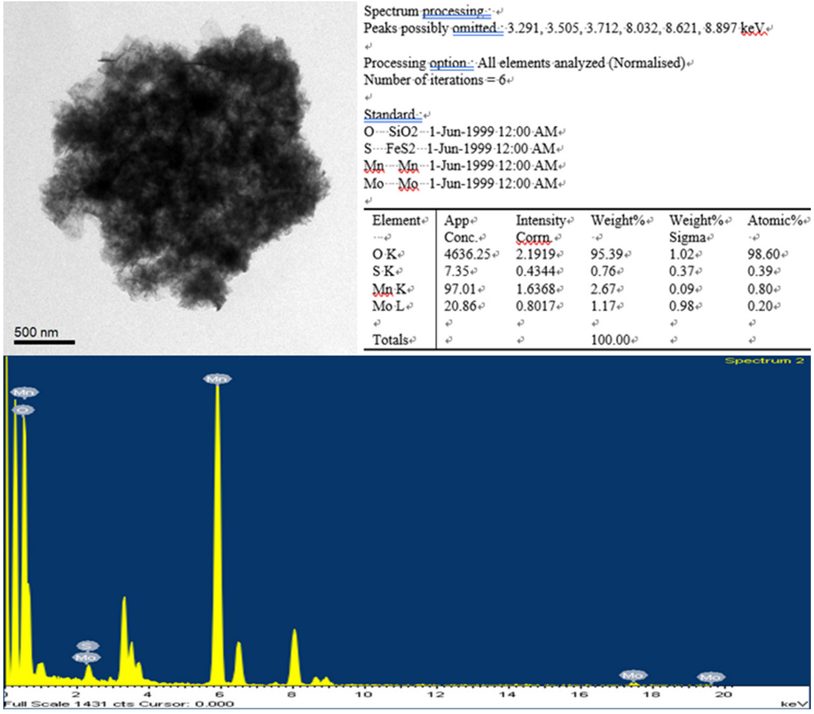

Spectrum processing :
Peaks possibly omitted : 3.291, 3.505, 3.712, 8.032, 8.621, 8.897 keV

Processing option : All elements analyzed (Normalised)
Number of iterations = 6

Standard :
O SiO2 1-Jun-1999 12:00 AM
S FeS2 1-Jun-1999 12:00 AM
Mn Mn 1-Jun-1999 12:00 AM
Mo Mo 1-Jun-1999 12:00 AM

Element	App Conc.	Intensity Corrn.	Weight%	Weight% Sigma	Atomic%
O K	4636.25	2.1919	95.39	1.02	98.60
S K	7.35	0.4344	0.76	0.37	0.39
Mn K	97.01	1.6368	2.67	0.09	0.80
Mo L	20.86	0.8017	1.17	0.98	0.20
Totals			100.00		

Figure S7.TEM energy dispersive analysis (EDS) image of the MnS/MoS_2 composite.

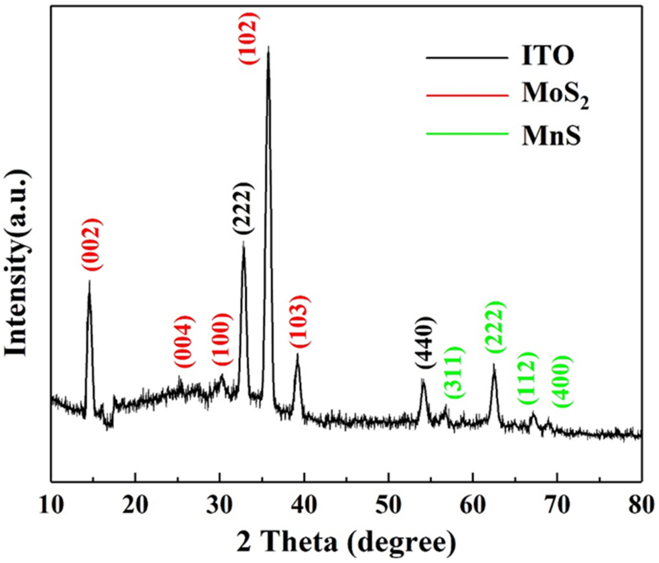

Figure S8. XRD diffraction patterns of MnS/MoS_2 composite thin films.

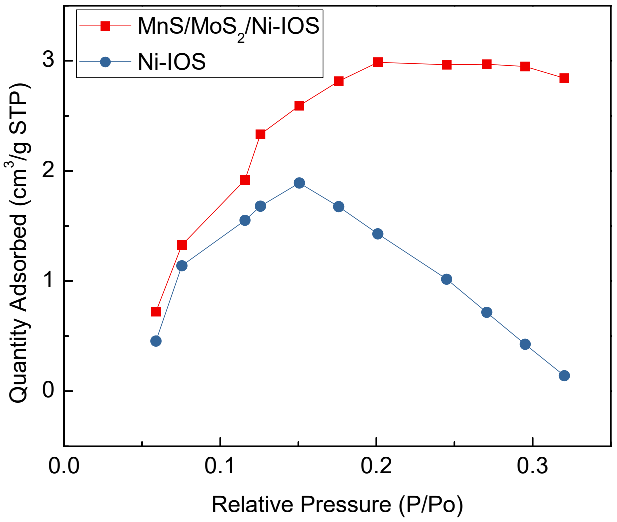

Figure S9. The BET adsorption curve of MnS/MoS2/Ni-IOS composite thin films.
